# Zero—Icebergs—Walrussia

**Published:** 1867-07

**Authors:** 


### ZERO—ICEBERGS—WALRUSSIA.

THE publishing house of HURD & HOUGHTON, 459 Broome Street, New-York, have issued a new volume, which will be a standard book of reference both at home and abroad, entitled, "The Open Polar Sea," being a narrative of a voyage of discovery toward the North Pole, in the schooner "United States," by Dr. I. I. HAYES, with a splendid engraving, and autograph of the author. 8vo, 454 pages. The thick white paper, the large, clear type, and the elegant binding are proofs of the taste and enterprise of the publishers. There are several engravings, sketched by Dr. Hayes himself, while the subjects treated are of enduring value, and are so graphically presented, that the book itself has the interest of a romance, with the advantage that every line is literally true. The volume has been so well received by the press and the public, that we doubt not of there being successive calls for new editions. Dr. Hayes, in the concluding lines, answers the "*cui bono*," what's the use of spending so much time and money in pushing investigations in such inhospitable climes, by responding, with great beauty and power, that science follows the discoverer, guiding, supporting, and instructing, to be followed by Christianity, and the two, moving hand in hand together, steadily unfold to the human understanding the material interests which concern this life, and to the human soul the sacred truths of Revelation, which concern the life to come.

